# The scientific African diaspora in the Nordic region: policy perspectives on strengthening Africa-Nordic technology collaboration

**DOI:** 10.3389/frma.2026.1815969

**Published:** 2026-08-10

**Authors:** Diana A. Afrifa, Andrea C. del Valle

**Affiliations:** 1 Organization for Women in Science for the Developing World (OWSD), Trieste, Italy; 2 Department of Physiotherapy, Metropolitan Hospital, Ghana Health Service, Cape Coast, Ghana; 3 Department of Laboratory Medicine, Karolinska Institutet, Huddinge, Sweden

**Keywords:** African scientific diaspora, capacity building, knowledge transfer, Nordic- African collaboration, science diplomacy, technology transfer

## Abstract

Technological transformation across African nations is progressing rapidly through digitalization, enhanced mobile connectivity, and the growth of local innovation ecosystems. However, enduring structural constraints, including limited absorptive capacity, infrastructural deficits, institutional fragility, and skills shortages, continue to impede the diffusion of innovation. Overcoming these systemic obstacles requires mobilising transnational knowledge networks and strengthening global science and innovation linkages. In this regard, the evolving bilateral collaboration between the Nordic countries and Africa presents a strategically significant opportunity for fostering inclusive innovation and sustainable development. Within this collaborative framework, the African scientific diaspora residing in the Nordic region occupies a pivotal position, given its deep integration within advanced research and innovation systems characterised by substantial public investment, technological sophistication, and a culture of international cooperation. Acting as both knowledge brokers and institutional bridge-builders, these researchers and innovators are uniquely positioned to align Nordic scientific capabilities with Africa's developmental priorities. Advancing this collaboration requires coherent policy mechanisms, institutional support structures, and long-term engagements that facilitate reciprocal knowledge exchange and promote innovation-led capacity building across both regions.

## 1 Introduction

Africa stands at a pivotal moment in its development trajectory, poised on the edge of a technological renaissance. Across the continent, expanding mobile connectivity, rapid digitalization, and an accelerating wave of entrepreneurial activity are reshaping socioeconomic landscapes (Kayser et al., 2023). Innovation hubs, start-ups, and data-driven enterprises are creating opportunities that were unimaginable a decade ago. Yet the continent's progress remains uneven. Deep-seated structural challenges, limited absorptive capacity, insufficient human capital, fragmented institutions, and fragile infrastructure continue to constrain Africa's full participation in the knowledge economy (Jauhiainen, 2023). Overcoming these limitations will require more than capital investment; it calls for intentional integration into the global architecture of science, technology, and innovation (STI).

In recent years, growing attention has been paid to the role of scientific diasporas in strengthening research collaboration, the exchange of innovation, and technology transfer between host and origin countries (Bonilla et al., 2022a). Scientific diasporas are increasingly viewed not only as migrant communities but also as transnational knowledge networks that can support development through mentorship, co-publications, institutional partnerships, and the diffusion of innovation (Dafallah, 2025). One of Africa's most potent yet underleveraged resources lies beyond its borders: the African scientific diaspora (Bonilla et al., 2022b,a). This globally dispersed community, often educated or professionally established in advanced research ecosystems, represents a reservoir of expertise, networks, and transnational experience. While remittances and diaspora entrepreneurship are widely acknowledged drivers of development, (Adams and Page, 2005) the intellectual and policy contributions of scientific diasporas to Africa's innovation systems have received far less institutional attention. Harnessing their capacity could redefine how the continent accesses cutting-edge research, adapts global technologies to local realities, and builds resilient, innovative ecosystems.

Among the global diaspora landscape, the scientific African communities residing in the Nordic countries (Sweden, Denmark, Finland, Norway, and Iceland) offer a distinctive and strategic opportunity. The Nordic region, consistently ranked among the world's strongest innovation economies and characterised by long-standing investments in research excellence, a tradition of trust-based governance, and collaborative innovation ecosystems, provides a stimulating environment for scientific advancement (Balashova and Abramova, 2021). These environments provide African scientists in the Nordics with access to advanced research ecosystems, global funding networks, and institutional partnerships that may support scientific collaboration with African institutions. African researchers integrated within these systems occupy a unique nexus: they are participants in some of the world's most innovative research enterprises while maintaining cultural and professional ties with African academic and policy institutions. This dual positioning enables them to act as natural intermediaries, capable of translating scientific knowledge across contexts and fostering North-South technological partnerships grounded in mutual benefit. Their engagement has also been supported by institutional mechanisms such as the Nordic Africa Institute, which explicitly seeks to build capacity in knowledge production on Africa and to promote relations between African and Nordic research communities through scholarship programs and strategic partnerships. In parallel, regional initiatives such as Southern African-Nordic Centre (SANORD)^1^ have helped consolidate academic cooperation across Nordic and Southern African institutions, creating platforms for research collaboration, exchange, and capacity development. Collectively, these developments underscore the role of the diaspora not only as a community of professionals abroad but also as a strategic intermediary in science diplomacy, knowledge circulation, and Africa-Nordic technological cooperation.

To unlock this potential, a deliberate policy framework is required: one that moves from *ad hoc* engagement to structured collaboration. Policymakers in both regions should establish formal mechanisms to institutionalise the scientific diaspora's involvement (Birka and Klavinš, 2020). This may include co-funded bilateral research programs focusing on shared priorities such as climate resilience, digital health, and sustainable energy; dedicated diaspora science networks that promote mentorship and mobility; and joint innovation funds that facilitate translational research linking Nordic expertise with African socioeconomic challenges (Jauhiainen, 2023; Hamro-Drotz and Brüning, 2015). Furthermore, universities, research councils, and development agencies could create fellowship schemes that encourage circular mobility, enabling African scholars in the Nordic region to contribute to capacity building at home without sacrificing their research continuity abroad.

By embedding these initiatives within broader science diplomacy efforts, governments can reimagine diaspora engagement as a cornerstone of transnational innovation strategy (Hoy, 2018). The scientific African diaspora is not merely a symbolic connection to the continent; it is an active bridge for knowledge transfer, institutional learning, and the co-creation of technology aligned with local needs. Realising Africa's technological ambitions, therefore, depends not only on strengthening innovation ecosystems within its borders but also on more effectively leveraging its global scientific community as co-architects of the continent's future.

Earlier discussions on migration and development largely framed the emigration of highly skilled professionals from Africa as “brain drain” (Bonilla et al., 2022b). More recent scholarship has shifted towards concepts such as “brain circulation,” “knowledge exchange,” and “diaspora engagement,” recognising that highly skilled migrants can contribute to development through remote collaboration, mentorship, institutional partnerships, and technology adaptation (Daugeliene and Marcinkeviciene, 2009). Scientific diasporas have therefore become increasingly relevant within global discussions on science diplomacy, innovation systems strengthening, and transnational research collaboration.

However, evidence from Africa remains fragmented. Existing literature often focuses on remittances, migration trends, or health workforce mobility rather than technology transfer and innovation systems (Frehywot and Park, 2019). There is also limited literature examining how African scientific diasporas in the Nordic region contribute specifically to technology transfer, research collaboration, and innovation capacity building across Africa.

This perspective article examines how the scientific African diaspora in the Nordic region can contribute to technology transfer and innovation capacity building across Africa. Drawing on policy analysis, research literature, and emerging examples of diaspora engagement, the paper argues that the Nordic-based African scientific diaspora represents a strategically positioned but underutilised transnational resource for strengthening Africa's STI systems. The paper further proposes analytical pathways and strategic recommendations for supporting sustained diaspora-led collaboration between African and Nordic institutions.

### 1.1 Conceptualising the diaspora as a strategic bridge for technology transfer and innovation

The term diaspora broadly refers to communities of people who are dispersed or scattered from their original homeland. Contemporary scholarship conceptualises diaspora not only as dispersion, but also as the continued maintenance of material, cultural, emotional, or professional ties with their homelands (Frehywot and Park, 2019). In recent years, the concept has evolved beyond its sociocultural roots to include a developmental aspect, recognising diasporas as transnational actors capable of promoting knowledge and technology transfer, research collaboration, and innovation-driven growth. Diaspora networks often serve as dynamic interfaces between advanced research settings in host countries and emerging innovation ecosystems in their countries of origin, facilitating reciprocal flows of knowledge, expertise, and technological resources (Echeverría-King et al., 2022).

In this paper, the term “scientific African diaspora” refers to African-born or African-descended scientists, researchers, engineers, clinicians, innovators, and technology professionals residing abroad who maintain professional or collaborative linkages with African institutions or research systems. The term is used in a functional rather than political sense. Scientific diaspora engagement has increasingly become part of discussions on global research collaboration and innovation policy. Earlier migration theories focused heavily on the negative consequences of skilled migration from low- and middle-income countries (Docquier and Rapoport, 2012). Over time, however, researchers began to recognise that highly skilled migrants often maintain active professional links with their countries of origin through research collaboration, mentorship, training, and institutional partnerships (Frehywot and Park, 2019). The concept of “brain circulation” emerged from this shift. Rather than viewing migration solely as a permanent loss of expertise, brain circulation highlights the movement of knowledge, skills, and innovation across borders through mobile scientific networks (Daugeliene and Marcinkeviciene, 2009).

Diaspora networks often function as bridges between advanced research environments and emerging innovation ecosystems in their countries of origin (Docquier and Rapoport, 2012). Knowledge and technology transfer through diaspora engagement may occur through multiple mechanisms: academic collaboration, co-publications, mentorship, return mobility, digital exchange, and institutional partnerships (Tijssen, 2007). These processes collectively build technological capabilities, promote contextually relevant innovation, (Del Valle, 2025) and strengthen institutions' absorptive capacity. For instance, the African scientific diaspora in the Nordics occupies a unique position within highly advanced STI systems, benefiting from robust research funding, interdisciplinary cooperation, and a culture of open innovation (Bonilla et al., 2022b). By leveraging these assets through formal and informal networks, this community can catalyze Africa's technological advancement and foster sustainable pathways for the transfer of innovation.

Importantly, translating these capabilities into tangible outcomes in African contexts requires an understanding of the structural and managerial challenges inherent in local ecosystems. As highlighted by African Scalecraft,^2^ scaling innovation in Africa is often constrained by limited absorptive capacity, weak infrastructure, fragmented institutional support, and gaps in financial and human capital. Effective technology transfer, therefore, demands more access to global knowledge through the diaspora. It calls for deliberate strategies to strengthen local innovation infrastructure, build organisational capacity, and adapt technologies to contextual realities. This reinforces the importance of integrating diaspora engagement into coherent policy frameworks and institutional mechanisms that align global expertise with local development needs.

The scientific diaspora is thus not only a source of remittances or philanthropy. It is an active channel for scientific exchange, knowledge sharing, and collaborative problem-solving. When strategically engaged, diaspora networks can complement continental efforts such as the African Union's Science, Technology, and Innovation Strategy for Africa (STISA-2024), which emphasises leveraging global partnerships to drive local development (Union, 2024). The African scientific diaspora in the Nordics, therefore, remains a vital yet underused link for co-developing solutions to the continent's most pressing technological challenges.

Within Africa, diaspora engagement has gained growing policy relevance. However, implementation gaps remain substantial. African universities and research institutions continue to face challenges related to funding instability, weak research infrastructure, limited digital systems, and administrative bottlenecks (Gaye et al., 2024). At the same time, evidence suggests that diaspora engagement is most effective when supported by strong institutional coordination, digital connectivity, and sustained policy support (Echeverría-King et al., 2022 Docquier and Rapoport, 2012). Structured collaboration platforms, mentorship programs, and institutional partnerships have been shown to strengthen scientific networking and promote cross-border knowledge exchange (Bonilla et al., 2021).

Despite these developments, there remains limited research examining the Nordic-based African scientific diaspora as a distinct case within the broader diaspora and innovation literature. This gap is important because Nordic countries possess strong innovation ecosystems, robust public research systems, and long-standing development partnerships with African institutions (Pandya et al., 2025; Balashova and Abramova, 2021). Understanding how diaspora scientists navigate and connect these systems may therefore provide useful insights for strengthening technology transfer and innovation collaboration across regions.

### 1.2 Enabling conditions for effective diaspora-technology bridging

Creating effective linkages between the scientific African diaspora and institutions on the continent requires an enabling ecosystem that supports collaboration, trust, and continuity. Several enabling conditions influence whether diaspora engagement translates into meaningful technology transfer and innovation collaboration. Structured platforms and digital portals that match diaspora expertise with specific institutional or national technology needs have proven effective in catalysing research and innovation linkages (Chand, 2016). Likewise, organised scientific diaspora networks and mentorship platforms have been shown to facilitate collaboration, networking, and knowledge exchange across borders. For example, initiatives such as the African Diaspora Policy Centre^3^ and the African Diaspora Network^4^ have demonstrated how curated matchmaking and mentorship programs can convert diaspora interest into tangible partnerships. These structures help reduce fragmentation and create clearer pathways for sustained engagement.

Institutional incentives also play an important role. Visiting fellowships, adjunct professorships, or secondment programs may encourage sustained diaspora participation by recognising professional contributions and reducing bureaucratic barriers (Tierney et al., 2013). Such arrangements may strengthen long-term institutional relationships while creating opportunities for skills transfer, mentorship, and collaborative innovation. Moreover, improved digital infrastructure and collaborative platforms within African universities and research centres facilitate remote supervision, co-publications, and technology transfer in an increasingly digital scientific environment (Frehywot and Park, 2019). Policies that promote intellectual property protection, knowledge sharing, and researcher mobility further strengthen mutual trust and lower risks in international collaboration.

Additionally, dedicated funding mechanisms are equally important. Seed grants, travel fellowships, innovation funds, and co-creation research financing schemes can help turn diaspora-led initiatives into practical innovations and local capacity-building. An example of such programs is the Nordic Africa Institute Scholarship Program, which provides travel, workspace, and subsistence support for Africa-focused researchers. Another example is the Nordic Development Fund Grant Financing, which co-finances projects that link Nordic expertise to African development priorities.^5^ These organisational funding mechanisms illustrate the practical ways in which Nordic institutions enable diaspora engagement in research and technology transfer. Without sustained financing, many collaborations remain short-term or dependent on individual relationships rather than institutional systems. Together, these enabling conditions lay the foundation for a resilient framework for diaspora-driven technological advancement.

### 1.3 Challenges and barriers to diaspora engagement in technology transfer

Despite growing interest in diaspora engagement, several structural and systemic challenges prevent effective technology bridging between the African scientific diaspora and the continent. Weak absorptive capacity within home-country institutions, characterised by limited research infrastructure, unstable funding, and administrative inefficiencies, remains a significant bottleneck that limits diaspora contributions (Gaye et al., 2024; Pulford et al., 2020). Incentive misalignment also remains an important barrier. Diaspora scientists may face limited institutional recognition, unclear engagement pathways, inadequate remuneration, and bureaucratic delays when attempting to collaborate with institutions in their countries of origin (Nwadiuko et al., 2016; Del Valle, 2023). In some cases, short-term collaborations rely heavily on personal motivation rather than structured institutional support, making long-term sustainability difficult.

Fragmentation across diaspora networks presents another challenge. African diasporas are diverse and often organised around national, linguistic, or professional identities rather than continental structures, operating in isolation without coherent coordination or policy frameworks (Nnabuihe et al., 2024; Pandya et al., 2025). As a result, collaboration efforts may remain scattered and poorly coordinated, limiting collective impact and reducing opportunities for large-scale technology partnerships.

Moreover, tensions between brain drain and brain gain persist; when domestic systems are unattractive, skilled professionals may hesitate to engage with or return to them, worsening the knowledge loss gap (Poppe et al., 2016). Communication, trust, and logistical barriers, including differences in time zones, institutional bureaucracy, and cultural expectations, often compound these difficulties (Tierney et al., 2013). Differences in administrative systems, research governance structures, digital capacity, and institutional expectations can slow joint projects and create uncertainty within partnerships. Furthermore, uneven political commitment to science and innovation across African countries may influence the extent to which diaspora engagement is prioritised within national development strategies.

Finally, there remains limited empirical evidence evaluating the long-term impact of diaspora-led technology transfer initiatives in Africa, leaving policy and practice primarily guided by anecdotal evidence (Tierney et al., 2013). Existing studies often focus on migration patterns or remittances rather than institutional innovation outcomes, technology adaptation, or research system strengthening. This evidence gap makes it difficult to identify best practices, evaluate effectiveness, or design scalable diaspora engagement models. These interconnected barriers highlight the need for coordinated policy frameworks and sustained investment to transform Africa's scientific diaspora from an underutilised resource into a strategic driver of technological advancement and innovation capacity building.

In response to these challenges, several European frameworks have begun integrating structured mechanisms to strengthen cross-regional collaboration and data-driven impact. The Horizon Europe-Africa Initiative III, for instance, allocates over €500.5 million to joint research consortia that link African institutions with European partners on themes such as climate resilience and digital innovation (Worldwide, 2025). Likewise, the Joint Innovation Facility (JIF) fosters co-funded research and development (R&D) that promotes technology localisation and equitable knowledge exchange.^6^ The EUREKA Network, with active participation from Nordic countries, complements these efforts by supporting international R&D calls that encourage joint projects, promote sustained partnerships, and deliver measurable outcomes and technology localisation.^7^ Together, these initiatives demonstrate how coordinated, well-funded, and monitored collaboration models can overcome many of the systemic barriers currently limiting diaspora-led technology transfer. Addressing these constraints is critical to converting Africa's vast diaspora potential into a sustained, measurable driver of technological transformation.

### 1.4 Strengthening science, technology, and innovation through diaspora-led collaborative partnerships

Several initiatives illustrate the growing role of scientific diasporas in strengthening research collaboration and innovation systems. Organised scientific diaspora networks in Latin America and the Caribbean have demonstrated how diaspora scientists can contribute to science diplomacy, mentorship, institutional networking, and collaborative research (Bonilla et al., 2021; Echeverría-King et al., 2022). Likewise, a scoping review found that diaspora engagement in health system strengthening frequently involves joint training, remote mentorship, and collaborative research (Cabrera and del Valle, 2025a). These activities effectively transfer tacit and technical knowledge to local institutions, yet structural and funding limitations frequently constrain their impact (Taslakian et al., 2022). Although these initiatives emerge from different regional contexts, they provide useful lessons for African diaspora engagement.

In the African context, collaborations between institutions have increasingly emphasised joint research, implementation science, digital health, and equitable partnership models. The African Diaspora Network^8^ (ADN) implements programs such as the *Builders of Africa's Future*, which connects African entrepreneurs and scientists in the diaspora with counterparts on the continent to co-develop scalable innovations in health, agriculture, and digital technology. Such initiatives demonstrate how targeted funding and mentorship structures can help translate diaspora expertise into tangible innovation ecosystems.

Similarly, the African Technology Policy Studies Network (ATPS)^9^ has institutionalised diaspora participation by creating formal chapters in the United States, United Kingdom, and Australia. These chapters act as knowledge bridges, encouraging African researchers abroad to contribute to national innovation policies, research collaborations, and science capacity-building efforts across Africa. Through policy-focused research and regional workshops, ATPS has facilitated joint technology foresight studies and supported science-policy dialogues that incorporate diaspora input into African national STI strategies.

Beyond Africa-based networks, the Africa-Europe Innovation Partnership (AEIP)^10^ connects innovation actors from both regions, including several Nordic research institutions and startups. The AEIP supports co-creation and technology matchmaking platforms that enable transcontinental partnerships and knowledge exchange between African entrepreneurs and European innovation hubs. The initiative has shown that when diaspora professionals are integrated into structured frameworks like AEIP, they can accelerate collaborative innovation, access to funding, and technology transfer between Europe and Africa.

### 1.5 Mobilising the scientific African diaspora in the Nordics

Mobilising the scientific African diaspora in the Nordic countries has significant potential to advance Africa's STI agenda. First, diaspora engagement can enhance human capital and brain circulation, counteracting the persistent challenge of talent outflows from the continent. Studies have shown that diaspora scientists contribute to “brain gain” by transferring skills, knowledge, and mentorship to home institutions through virtual collaborations and visiting scholar programs (Hofman and Kramer, 2015). The global networks and access embedded in Nordic research ecosystems, characterised by strong international collaborations and open science practices, position African scientists as vital intermediaries. They connect African institutions to global funding, technologies, and research consortia.

Initiatives such as the Nordic Africa Institute have promoted collaborations that adapt Nordic digital health and renewable energy innovations to African contexts. On the innovation ecosystem side, events like the maiden Nordic-Africa Startup Summit at *TechBBQ* in Copenhagen, Denmark, bring together Nordic and African startups, investors, universities, and ecosystem leaders.^11^ Participants collaborate across agrotech, clean tech, fintech, and life sciences, fostering co-creation and localisation of technology. Similarly, the Norwegian African Business Association's Nordic-African Business Summit convenes African entrepreneurs and Nordic investors to explore opportunities for scale-up and joint innovation initiatives.^12^

Recent Nordic policy frameworks further strengthen this bridge. Finland, Denmark, and Norway have each adopted new Africa strategies that prioritise equitable, innovation-led partnerships, trade, investment, and the green transition (Boafo et al., 2025; Chiteka and Enweremadu, 2025; Qamruzzaman and Karim, 2024). These strategies mark a shift away from purely aid-driven models. They emphasise multilateral cooperation, African-led solutions, and a stronger African voice in global forums. Sweden engages Africa through targeted bilateral and regional initiatives, often implemented in partnership with the Swedish International Development Cooperation Agency (SIDA).^13^ These initiatives support Africa-focused research programs, mobility schemes, and capacity-building efforts that link Swedish universities and research institutions with African partners. Meanwhile, Iceland is developing its first comprehensive Africa strategy,^14^ signalling an emerging commitment to structured, continent-wide engagement that could provide new opportunities for Nordic-African scientific collaboration. Finally, the Goalkeepers event, held for the first time in 2026 in the Nordics by the Gates Foundation, sent a strong message to the world and the Nordic business about the need for continued investment in Africa for future generations.^15^

Nevertheless, concrete academic partnerships illustrate how these strategies translate into actionable knowledge exchange. A prime example is the long-standing collaboration between Karolinska Institutet (KI) in Sweden and Makerere University in Uganda. This partnership includes joint doctoral training, student and faculty exchanges, and the creation of a Centre of Excellence for Sustainable Health (CESH).^16^ Through capacity-building and research programs in maternal health, infectious disease control, and health systems strengthening, this alliance exemplifies how knowledge transfer can be institutionalised within North-South academic collaborations.

### 1.6 Perspectives

**Figure 1:**
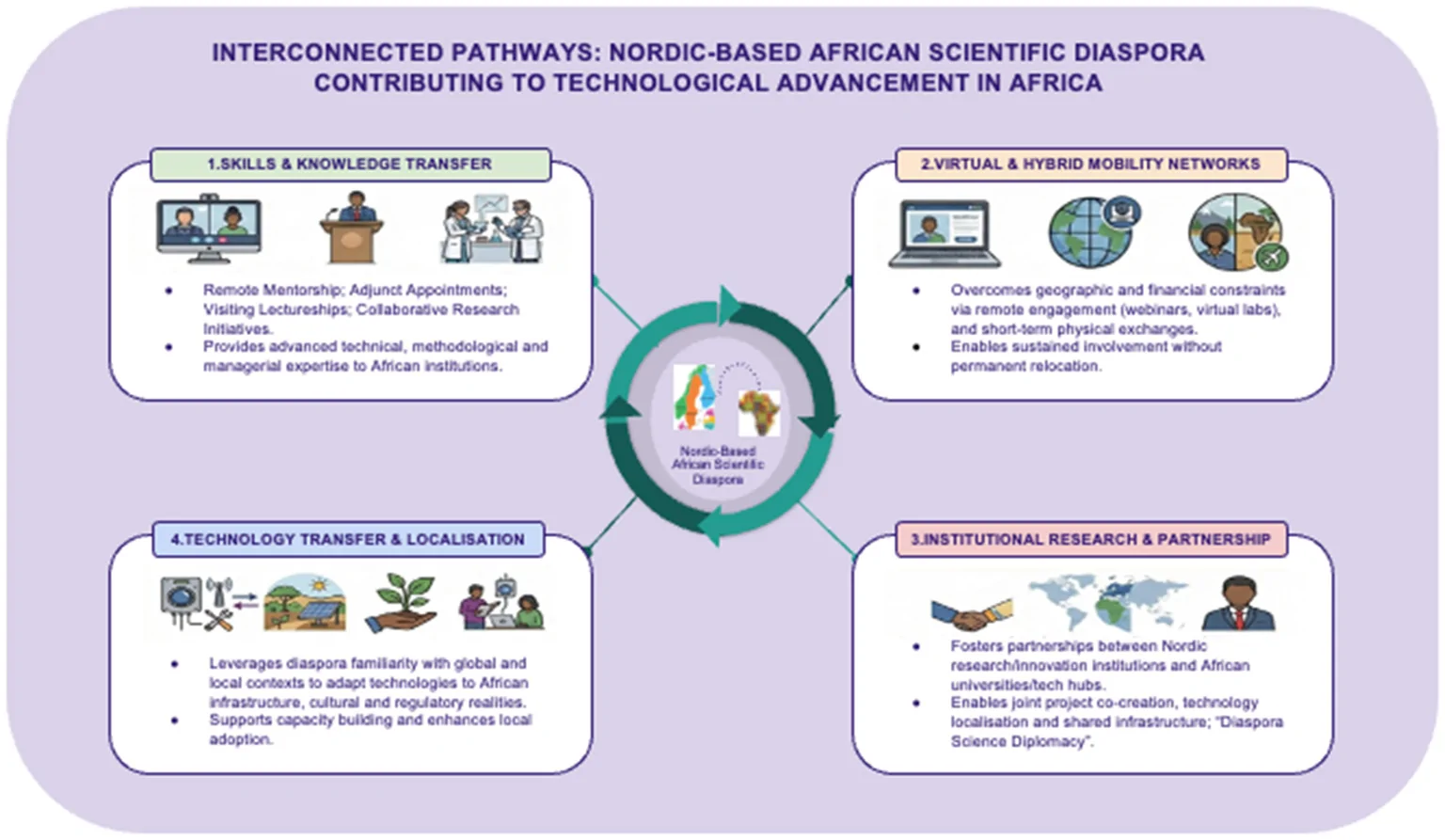
Interconnected pathways for mobilizing the scientific African diaspora in the Nordics to advance technology in Africa.

This paper proposes four interconnected pathways through which the scientific African diaspora in the Nordic region can make substantial contributions to technological advancement in Africa (Figure 1).

#### 1.6.1 Skills and knowledge transfer

Skills and knowledge transfer take place as diaspora scientists and innovators share advanced technical, methodological, and managerial expertise with African institutions. They do this through remote mentorship, adjunct appointments, visiting lectureships, and collaborative research initiatives (Ng'oda et al., 2024; del Valle, 2025). These activities support both individual skills development and institutional research capacity. Knowledge transfer is particularly important in specialised fields where many African institutions continue to face shortages of technical expertise and advanced research infrastructure.

However, effective knowledge transfer depends on institutional readiness. Universities and research centres require stable funding systems, protected research time, digital infrastructure, and administrative support to sustain long-term collaboration. Without these conditions, knowledge exchange may remain fragmented and difficult to institutionalise.

#### 1.6.2 Virtual and hybrid mobility networks

Virtual and hybrid mobility networks help overcome geographic and financial constraints. Digital collaboration has expanded opportunities for diaspora engagement beyond permanent return migration. They facilitate remote engagement through webinars, virtual laboratories, and short-term physical exchanges, increasingly allowing scientists to contribute across borders while remaining embedded within host-country institutions. These models enable sustained diaspora involvement without requiring permanent relocation. An example of such an initiative is the Nordic Africa Institute's African Scholarship Program. It enables researchers based in Africa to visit a Nordic host institution for up to 3 months, fostering academic exchange and capacity building without requiring permanent relocation. In a similar vein, the *MIDWIZE* initiative under CESH combines webinars, virtual modules, and short-term on-site training to enhance the skills of midwives and health officials in Uganda. *MIDWIZE* is a midwife-led quality improvement initiative that aims to improve maternal and neonatal outcomes through evidence-based practices and collaborative learning (Lindgren and Erlandsson, 2022). Such structured hybrid models illustrate how effective knowledge transfer and capacity building can be achieved while minimising the need for permanent relocation. These approaches may reduce financial barriers and increase flexibility for diaspora engagement. However, digital inequality remains a major concern. Uneven internet connectivity, limited digital infrastructure, and restricted access to collaborative technologies continue to affect many institutions across Africa (Pulford et al., 2020). As a result, virtual engagement models must be accompanied by investment in digital systems and institutional support structures.

#### 1.6.3 Institutional research and partnerships

Diaspora scientists may also serve as institutional brokers, connecting Nordic research and innovation institutions with African universities and technology hubs. Through professional networks and institutional affiliations, they foster joint project co-creation, support localisation of technology, and promote the development of shared research infrastructure. This role aligns with the emerging concept of “diaspora science diplomacy,” in which diaspora scientists advocate for and facilitate science and technology cooperation for Africa's benefit. A notable example is the previously mentioned *MIDWIZE* initiative, in which researchers from Karolinska Institutet in Sweden and Makerere University in Uganda collaborated to co-develop midwife-led quality-improvement interventions (Blomgren et al., 2024). This partnership leveraged Nordic expertise to strengthen local institutional capacities and implement contextually relevant solutions that improved maternal and newborn health outcomes (Blomgren et al., 2025). While beneficial, it is important to note that institutional partnerships require careful management of power dynamics, authorship equity, resource distribution, and governance structures to ensure sustainable and equitable collaboration.

#### 1.6.4 Technology transfer and localisation

Technology transfer involves more than simply moving technologies from one setting to another. Successful implementation often requires adaptation to local infrastructure, workforce realities, cultural contexts, regulatory systems, and financial constraints (Heeks, 2002). Technology transfer and localisation draw on the diaspora's deep understanding of both global innovation systems and African socio-economic contexts. This dual familiarity helps adapt technologies to local infrastructure, culture, and regulations. In turn, it strengthens capacity building and promotes local adoption. This pathway is particularly relevant in fields such as digital health, clean energy, artificial intelligence, diagnostics, and biomedical innovation. Nevertheless, technology transfer alone cannot produce sustainable innovation without parallel investment in local manufacturing capacity, regulatory systems, workforce development, and institutional research ecosystems.

Together, these pathways show that diaspora engagement is far more than entrepreneurship or financial contribution. It is a multifaceted process that drives capability building, network strengthening, innovation localisation, and the cultivation of sustainable knowledge ecosystems. Building on these pathways, the scientific diaspora can also be conceptualised as a strategic bridge connecting global research ecosystems with African innovation hubs, as highlighted in recent analyses of transnational migration and international business networks (Mockaitis, 2023). This perspective emphasises circular and hybrid mobility models in which diaspora scientists engage through short-term visits, virtual collaborations, and remote mentorship, enabling sustained involvement without permanent relocation. By facilitating institutional co-creation, knowledge exchange, and technology adaptation, these hybrid structures reinforce the diaspora's role as a catalyst for locally relevant innovation and cross-regional development. In addition, cost-effective engagement models, such as hybrid mentorships and online incubator programs, have shown great success in mobilising expertise. These approaches eliminate the high costs associated with physical infrastructure and large-scale facility investments (Schouw et al., 2025). Such efforts also align with Agenda 2063 and the Sustainable Development Goals (SDGs), which both highlight STI as key drivers of sustainable development and inclusive growth (Africa's sustainable growth hinges on these). The Nordic-based African scientific diaspora therefore serves as a strategic bridge, fostering equitable global partnerships and accelerating Africa's innovation-led transformation.

### 1.7 Strategic recommendations

**Figure 2:**
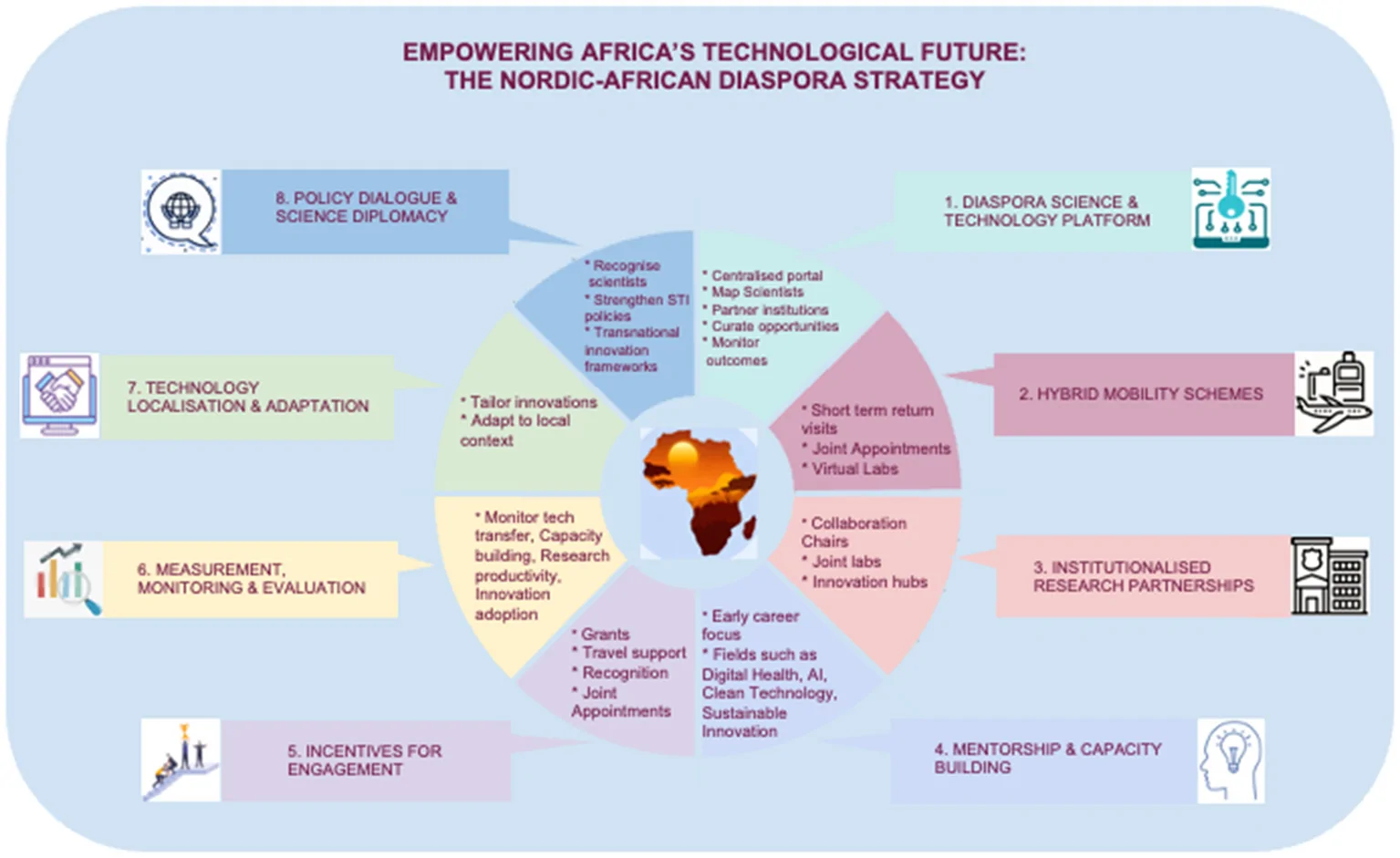
A comprehensive conceptual model outlining eight interconnected strategies to strategically engage the Nordic-based African scientific diaspora for technological transformation in Africa. Each element, from establishing a dedicated Nordic-African diaspora science technology platform to promoting policy dialogue, represents an actionable policy lever that operationalizes the diaspora's potential as a knowledge broker and institutional bridge builder.

To harness the potential of the Nordic-based African scientific diaspora for advancing technology in Africa, several strategic recommendations are proposed (Figure 2). Figure 2 presents a comprehensive conceptual model delineating eight interconnected strategies for systematically engaging the Nordic-based African scientific diaspora to drive technological transformation across Africa. These strategies, from establishing dedicated platforms and hybrid mobility schemes to institutionalised partnerships, targeted mentorship programs, technology localisation frameworks, incentive structures, evaluation mechanisms, and policy dialogue initiatives, collectively constitute a multilevel policy architecture that operationalizes the diaspora's potential as knowledge brokers and institutional intermediaries. The model addresses core structural barriers, including network fragmentation, incentive misalignment, and absorptive capacity limitations, while aligning local institutional capabilities with national and bilateral priorities.

#### 1.7.1 Diaspora science & technology platform

Establishing a dedicated Nordic-African Diaspora STI Platform will provide a central portal for mapping diaspora scientists, identifying partner institutions in Africa, curating collaborative opportunities, and monitoring engagement outcomes. Such platforms may improve coordination and reduce fragmentation across diaspora initiatives. However, long-term sustainability would require institutional ownership, stable funding, and transparent governance structures.

#### 1.7.2 Hybrid mobility schemes

Hybrid mobility schemes, including short-term return visits, virtual laboratories, and joint academic appointments between Nordic and African institutions, may enable active diaspora participation without requiring permanent relocation. These models may also support collaborative supervision, mentorship, and technology exchange. Nevertheless, implementation requires supportive visa policies, institutional agreements, and reliable digital infrastructure.

#### 1.7.3 Institutionalised research partnerships

Nordic-African collaboration chairs, joint laboratories, and innovation hubs may strengthen long-term institutional collaboration. There is a need for local governance mechanisms in Nordic countries, such as institutional review boards at universities like Karolinska Institutet, national research councils (e.g., Sweden's Vetenskapsrådet), and diaspora-led networks, to play a critical role in enabling African researchers to engage in R&D activities that bridge continents. These structures provide funding opportunities, ethical oversight, and collaborative platforms that support diaspora initiatives in areas like health innovation and sustainable technologies.

At the central level, Nordic government strategies (e.g., Sweden's SIDA partnerships, Norway's Africa-focused policies) align with African Union frameworks like STISA-2024, creating multilevel policy environments that facilitate technology transfer and equitable partnerships. These governance dynamics both enable and constrain diaspora contributions, highlighting the need for coordinated mechanisms that enhance policy alignment, reduce bureaucratic barriers, and amplify Africa-Nordic technological collaboration for sustainable development.

#### 1.7.4 Mentorship and capacity building

Equally important is the institutionalisation of Nordic-African research partnerships through collaboration chairs, joint laboratories, and innovation hubs that explicitly integrate diaspora scientists as bridge-builders. Diaspora-led mentorship programs may strengthen scientific leadership and technical skills among early-career African researchers. Mentorship and capacity-building programs targeting early-career African researchers in cutting-edge fields like digital health, (del Valle, 2025) artificial intelligence, clean technology, and sustainable innovation would enhance skills transfer and knowledge dissemination (Union, 2024). Such initiatives are particularly important in emerging fields, including digital health, clean technology, implementation science, and data science. Long-term mentorship structures may also strengthen research retention and institutional continuity.

#### 1.7.5 Incentives for engagement

Creating incentives, including research grants, fellowship schemes, travel support, recognition awards, and joint appointment opportunities, can further encourage diaspora-led initiatives supported by both African and Nordic governments and institutions. Institutional incentives can help transform diaspora engagement from *ad hoc* collaboration into structured long-term partnerships. However, funding systems must remain transparent and aligned with national research priorities.

#### 1.7.6 Measurement, monitoring, and evaluation

Robust measurement and evaluation frameworks are also essential to monitor diaspora contributions to technology transfer, capacity building, research productivity, and innovation adoption, ensuring accountability and guiding future initiatives. Investments in connectivity, digital laboratories, and collaborative platforms can improve data capture and analysis for assessing transnational scientific engagement. However, effective measurement, monitoring, and evaluation also require institutional capacity building, including training and technical support to ensure data quality and usability.

#### 1.7.7 Technology localisation and adaptation

Emphasising technology localisation and adaptation encourages diaspora scientists to tailor innovations, from software to clean energy solutions, to local infrastructural, cultural, and regulatory contexts. Policies that support local adaptation of technologies may improve sustainability and implementation outcomes. Diaspora scientists can help align innovations with local regulatory systems, infrastructure realities, and workforce needs. These localisation and adaptation efforts require stronger local manufacturing systems, regulatory institutions, and implementation capacity.

#### 1.7.8 Policy dialogue and science diplomacy

Promoting policy dialogue and recognising diaspora scientists as active players in science diplomacy strengthen local innovation policies, build global research connexions, and reinforce transnational innovation frameworks. Structured dialogue between governments, universities, funding agencies, and diaspora networks may strengthen policy alignment and collaborative governance. That said, political commitment and institutional coordination remain critical for sustaining these efforts.

Together, these measures form a comprehensive and actionable policy blueprint to mobilise the Nordic-based African scientific diaspora as a transformative force for Africa's technological development.

## 2 Conclusion

The Nordic-based African scientific diaspora represents an influential yet under-recognised conduit for accelerating technological advancement across the African continent. By leveraging their expertise, professional networks, and strategic institutional positions, this diaspora can significantly enhance technology transfer, strengthen human and institutional capacity, and promote innovation that is tailored to local contexts. Realising this potential requires establishing deliberate platforms, enabling policies, robust digital infrastructure, hybrid mobility frameworks, and rigorous impact evaluation mechanisms. Despite its potential, diaspora engagement alone is insufficient to address deeper structural weaknesses within African innovation systems. Sustainable impact depends on institutional absorptive capacity, long-term investment, digital infrastructure, equitable partnerships, and coherent governance systems. The Nordic-African case therefore offers broader lessons on how transnational scientific networks can support innovation-driven development when embedded within strong institutional and policy environments. As African nations chart their digital futures and STI agendas, engaging the Nordic-based African scientific diaspora should be viewed not as a peripheral effort but as a central pillar in building a resilient, innovation-driven Africa with sustainable technological capability.

## Data Availability

The original contributions presented in the study are included in the article/supplementary material, further inquiries can be directed to the corresponding author.

## Footnotes

1 SANORD: https://sanord.uwc.ac.za.

2 African Scalecraft: https://www.africanscalecraft.com.

3 African Diaspora Policy Centre: https://www.diaspora-centre.org.

4 African Diaspora Network: https://africandiasporanetwork.org.

5 Nordic Development Fund: https://www.ndf.int.

6 Spark Project Africa: https://project-spark.africa.

7 Eureka Project: https://www.eurekanetwork.org.

8 The African Diaspora Network: https://africandiasporanetwork.org.

9 African Technology Policy Studies Network (ATPS): https://atpsnet.org.

10 Africa-Europe Innovation Partnership (AEIP): https://www.africaeuropeinnovation.net.

11
https://techbbq.dk/nordicafrica-startupsummit

12
https://summit.norwegianafrican.no

13 Swedish International Development Cooperation Agency: https://www.sida.se.

14 https://nai.uu.se.

15 Gates Foundation Goalkeepers: https://www.gatesfoundation.org.

16 Centre of Excellence for Sustainable Health: https://cesh.health.
